# Crystal structure of Brinzolamide: a carbonic anhydrase inhibitor

**DOI:** 10.1107/S2056989016006022

**Published:** 2016-04-15

**Authors:** Huirong Zheng, Benyong Lou

**Affiliations:** a Department of Chemistry and Chemical Engineering, Minjiang University, Fuzhou 350108, People’s Republic of China

**Keywords:** crystal structure, Brinzolamide, carbonic anhydrase inhibitor, sulfonamide, thia­zine, absolute configuration, hydrogen bonding

## Abstract

In crystal structure of the title compound, C_12_H_21_N_3_O_5_S_3_ [systematic name: (*R*)-4-ethyl­amino-2-(3-meth­oxy­prop­yl)-3,4-di­hydro-2*H*-thieno[3,2-*e*][1,2]thia­zine-6-sulfonamide 1,1-dioxide], there exist three kinds of hydrogen-bonding inter­actions. The sulfonamide group is involved in hydrogen bonding with the secondary amine and the meth­oxy O atom, resulting in the formation of layers parallel to the *bc* plane. The layers are linked by an N—H⋯O hydrogen bond involving a sulfonamide O atom as acceptor and the secondary amine H atom as donor, which gives rise to the formation of a unique bilayer structure. The absolute structure of the mol­ecule in the crystal was determined by resonant scattering [Flack parameter = 0.01 (4)].

## Abstract 2

In the crystal structure of Brinzolamide, the various hydrogen bonds present lead to the formation of a bilayer structure. The absolute configuration of the asymmetric C atom was determined to be *R* by resonant scattering.

## Chemical context   

The crystal structures of organic solids are dominated mainly by hydrogen-bonding inter­actions (Steiner, 2002 ▸). Hydrogen bonding plays a crucial role in polymorphism of active pharmaceutical ingredients (Vippagunta *et al.*, 2001 ▸). Brinzolamide (Conrow *et al.*, 1999 ▸), is a carbonic anhydrase inhibitor used for the treatment of open-angle glaucoma or ocular hypertension (March & Ochsner, 2000 ▸). Herein,we report on the crystal structure of Brinzolamide and the hydrogen-bonding inter­actions present in the crystal packing.
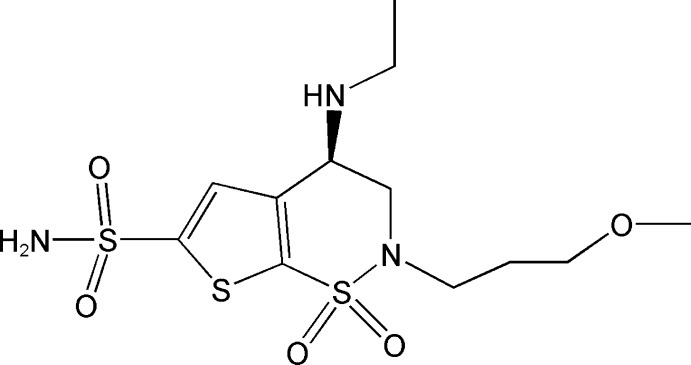


## Structural commentary   

**Figure 1:**
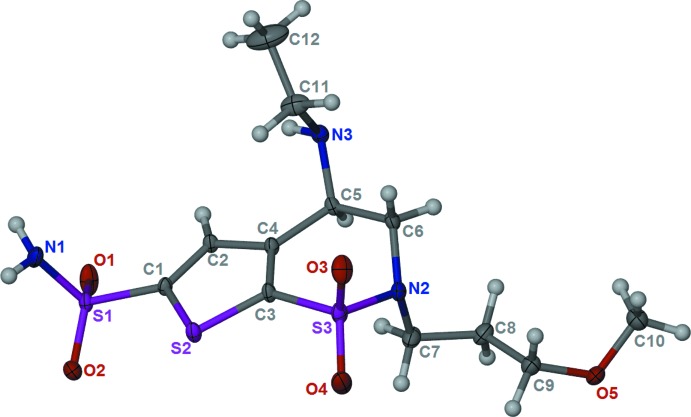
The mol­ecular structure of the title compound, showing the atom labelling and 30% displacement ellipsoids.

The mol­ecular structure of the title compound is shown in Fig. 1 ▸. The six-membered thia­zine ring has an envelope conformation with the N atom, N2, as the flap. The 3-meth­oxy­propyl chain has a twisted conformation with torsion angles N2—C7—C8—C9, C7—C8—C9—O5 and C8—C9—O5—C10 being 71.66 (18), 166.76 (14) and 82.04 (19)°, respectively. The ethyl­amino group (N3/C11/C12) is normal to the mean plane of the five planar atoms of the thia­zine ring (S3/C3–C6), making a dihedral angle of 84.4 (3)°. The three main functional groups (the sulfonamide, the secondary amine and the meth­oxy group) extend themselves in different directions, which facilitates the formation of a hydrogen-bonded network.

## Supra­molecular features   

**Figure 2:**
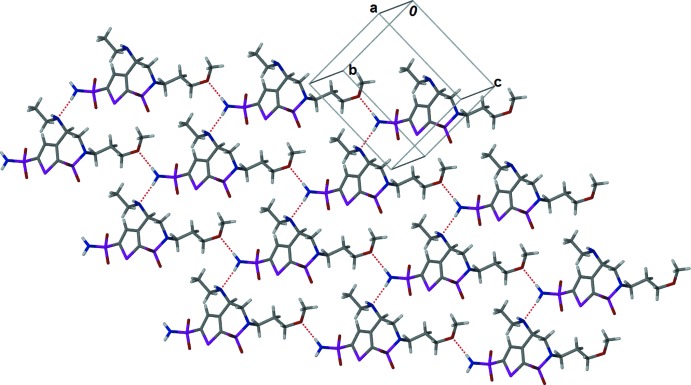
A view along the *a* axis of the two-dimensional hydrogen-bonded network in the crystal of the title compound. The hydrogen bonds are shown as dashed lines (see Table 1 ▸ for details).

**Figure 3:**
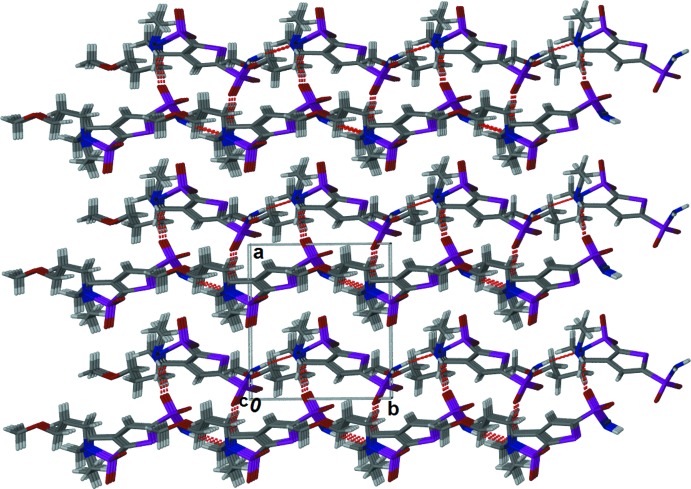
A view along the *c* axis of the crystal packing of the title compound, showing the hydrogen bonded bilayer structure. The hydrogen bonds are shown as dashed lines (see Table 1 ▸ for details).

There are three kinds of hydrogen-bonding inter­actions in the crystal of Brinzolamide (Table 1 ▸ and Figs. 2 ▸ and 3 ▸). The sulfonamide group is involved in hydrogen bonding [N1⋯N3 = 2.886 (2) Å, Table 1 ▸] with the secondary amine, forming a *C*(8) chain along the *b-*axis direction. The sulfonamide group is also involved in hydrogen bonding with the meth­oxy group [N1⋯O5 = 2.841 (2) Å, Table 1 ▸], linking the chains to form sheets parallel to the *bc* plane (Fig. 2 ▸ and Table 1 ▸). There also exists another hydrogen bond between the sulfonamide and the secondary amine [N3⋯O1 = 3.042 (2) Å, Table 1 ▸], linking the sheets to form a unique bilayer structure (Fig. 3 ▸).

## Database survey   

A search of the Cambridge Structural Database (CSD, Version 5.37, last update February 2016; Groom *et al.*, 2016 ▸) revealed no hits for Brinzolamide. A search for the fused six- and five-membered ring system, *viz.* 3,4-di­hydro-2λ^2^-thieno[3,2-*e*][1,2]thia­zine 1,1-dioxide, gave only two hits: 8b-bromo-2-(bromo­meth­yl)-4-methyl-3a-phenyl-1,3a,4,8b-tetra­hydro-2*H-*furo[2,3-*c*]thieno[3,2-*e*][1,2]thia­zine 5,5-dioxide (BUFQIE; Barange *et al.*, 2014 ▸) and (*S*)-6,6-dimethyl-4a,5,6,7-tetra­hydro-4*H*-pyrrolo­[1,2-*b*]thieno[3,2-*e*][1,2]thia­zine 9,9-dioxide (BUXDEE; Zeng & Chemler, 2007 ▸). The latter crystallizes in the chiral monoclinic space group *P*2_1_, with four independent mol­ecules in the asymmetric unit. However, in both compounds the six-membered thia­zine ring is also fused to a second five-membered ring; a tetra­hydro­furo ring in the case of BUFQIE, fused to the C—C bond, and a pyrrolo ring in the case of BUXDEE, fused to the N—C bond. The thia­zine ring in BUFQIE has a distorted twist-boat conformation, while in BUFQIE all four independent mol­ecules have half-chair conformations. This is in contrast to the situation in the title compound where the thia­zine ring has an envelope conformation with the N atom as the flap.

## Synthesis and crystallization   

The enanti­oselective synthesis of Brinzolamide has been reported by Conrow *et al.*, (1999 ▸). It is marketed under the trade name of Azopt by Alcon Laboratories, Inc., Fort Worth, Texas 76134, USA. Colourless prismatic crystals of Brinzolamide (383 mg, 1 mmol) were obtained by slow evaporation of a solution in chloro­form (15 ml).

## Refinement   

Crystal data, data collection and structure refinement details are summarized in Table 2 ▸. The NH and NH_2_ H atoms were located in difference Fourier maps and refined with distance restraints of N—H = 0.87 (1) Å for NH and 0.86 (1) Å for NH_2_ H atoms. The C-bound H atoms were included in calculated positions and treated as riding atoms: C—H = 0.95–1.00 Å with *U*_iso_(H) = 1.5*U*_eq_(C-meth­yl) and 1.2*U*_eq_(C) for other H atoms. The absolute structure of the mol­ecule in the crystal was determined by resonant scattering [Flack parameter = 0.01 (4)].

## Supplementary Material

Crystal structure: contains datablock(s) I, global. DOI: 10.1107/S2056989016006022/su5292sup1.cif

Structure factors: contains datablock(s) I. DOI: 10.1107/S2056989016006022/su5292Isup2.hkl

Supporting information file. DOI: 10.1107/S2056989016006022/su5292Isup3.cml

CCDC reference: 1473394

Additional supporting information:  crystallographic information; 3D view; checkCIF report

## Figures and Tables

**Table 1:** Hydrogen-bond geometry (Å, °)

*D*—H⋯*A*	*D*—H	H⋯*A*	*D*⋯*A*	*D*—H⋯*A*
N1—H1*B*⋯O5^i^	0.87 (1)	1.98 (1)	2.841 (2)	177 (2)
N1—H1*A*⋯N3^ii^	0.87 (1)	2.03 (1)	2.886 (2)	171 (2)
N3—H3⋯O1^iii^	0.86 (1)	2.26 (1)	3.042 (2)	151 (2)

Symmetry codes: (i) 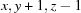; (ii) 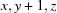; (iii) 
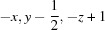
.

**Table 2:** Experimental details

Crystal data	
Chemical formula	C_12_H_21_N_3_O_5_S_3_
*M*_r_	383.50
Crystal system, space group	Monoclinic, *P*2_1_
Temperature (K)	293
*a*, *b*, *c* (Å)	9.698 (2), 8.8127 (19), 10.133 (2)
β (°)	92.248 (3)
*V* (Å^3^)	865.4 (3)
*Z*	2
Radiation type	Mo *K*α
μ (mm^−1^)	0.46
Crystal size (mm)	0.35 × 0.35 × 0.20

Data collection	
Diffractometer	Rigaku Mercury CCD
Absorption correction	Multi-scan (*CrystalClear*; Rigaku, 2000 ▸)
*T*_min_, *T*_max_	0.853, 0.913
No. of measured, independent and observed [*I* > 2σ(*I*)] reflections	6608, 3684, 3612
*R*_int_	0.010
(sin θ/λ)_max_ (Å^−1^)	0.649

Refinement	
*R*[*F* ^2^ > 2σ(*F* ^2^)], *wR*(*F* ^2^), *S*	0.022, 0.059, 1.04
No. of reflections	3684
No. of parameters	222
No. of restraints	4
H-atom treatment	H atoms treated by a mixture of independent and constrained refinement
Δρ_max_, Δρ_min_ (e Å^−3^)	0.21, −0.19
Absolute structure	1595 Friedel pairs; Flack (1983 ▸)
Absolute structure parameter	0.01 (4)

Computer programs: *CrystalClear* (Rigaku, 2000 ▸), *SHELXS97* and *SHELXL97* (Sheldrick, 2008 ▸), *X-SEED* (Barbour, 2001 ▸) and *PLATON* (Spek, 2009 ▸).

## supplementary crystallographic information

### Crystal data

**Table:**

C_12_H_21_N_3_O_5_S_3_	*F*(000) = 404
*M**_r_* = 383.50	*D*_x_ = 1.472 Mg m^−^^3^
Monoclinic, *P*2_1_	Mo *K*α radiation, λ = 0.71073 Å
Hall symbol: P 2yb	Cell parameters from 2619 reflections
*a* = 9.698 (2) Å	θ = 2.1–27.5°
*b* = 8.8127 (19) Å	µ = 0.46 mm^−^^1^
*c* = 10.133 (2) Å	*T* = 293 K
β = 92.248 (3)°	Prism, colourless
*V* = 865.4 (3) Å^3^	0.35 × 0.35 × 0.20 mm
*Z* = 2	

### Data collection

**Table:**

Rigaku Mercury CCD diffractometer	3684 independent reflections
Radiation source: fine-focus sealed tube	3612 reflections with *I* > 2σ(*I*)
Graphite monochromator	*R*_int_ = 0.010
Detector resolution: 14.6306 pixels mm^-1^	θ_max_ = 27.5°, θ_min_ = 2.0°
CCD_Profile_fitting scans	*h* = −12→12
Absorption correction: multi-scan (*CrystalClear*; Rigaku, 2000)	*k* = −11→11
*T*_min_ = 0.853, *T*_max_ = 0.913	*l* = −13→13
6608 measured reflections	

### Refinement

**Table:**

Refinement on *F*^2^	Secondary atom site location: difference Fourier map
Least-squares matrix: full	Hydrogen site location: inferred from neighbouring sites
*R*[*F*^2^ > 2σ(*F*^2^)] = 0.022	H atoms treated by a mixture of independent and constrained refinement
*wR*(*F*^2^) = 0.059	*w* = 1/[σ^2^(*F*_o_^2^) + (0.0403*P*)^2^ + 0.0611*P*] where *P* = (*F*_o_^2^ + 2*F*_c_^2^)/3
*S* = 1.03	(Δ/σ)_max_ = 0.001
3684 reflections	Δρ_max_ = 0.21 e Å^−^^3^
222 parameters	Δρ_min_ = −0.19 e Å^−^^3^
4 restraints	Absolute structure: 1595 Friedel pairs; Flack (1983)
Primary atom site location: structure-invariant direct methods	Absolute structure parameter: 0.01 (4)

### Special details

**Table:**

Geometry. All esds (except the esd in the dihedral angle between two l.s. planes) are estimated using the full covariance matrix. The cell esds are taken into account individually in the estimation of esds in distances, angles and torsion angles; correlations between esds in cell parameters are only used when they are defined by crystal symmetry. An approximate (isotropic) treatment of cell esds is used for estimating esds involving l.s. planes.
Refinement. Refinement of F^2^ against ALL reflections. The weighted R-factor wR and goodness of fit S are based on F^2^, conventional R-factors R are based on F, with F set to zero for negative F^2^. The threshold expression of F^2^ > 2sigma(F^2^) is used only for calculating R-factors(gt) etc. and is not relevant to the choice of reflections for refinement. R-factors based on F^2^ are statistically about twice as large as those based on F, and R- factors based on ALL data will be even larger.

### Fractional atomic coordinates and isotropic or equivalent isotropic displacement parameters (Å^2^)

**Table:**

	*x*	*y*	*z*	*U*_iso_*/*U*_eq_	
S1	0.08557 (3)	0.95125 (4)	0.63688 (3)	0.02705 (9)	
S2	0.26509 (4)	0.81437 (4)	0.85525 (3)	0.02964 (9)	
S3	0.37674 (4)	0.52417 (4)	0.99143 (4)	0.03230 (9)	
O1	−0.02692 (13)	0.88932 (16)	0.55817 (13)	0.0482 (3)	
O2	0.05935 (13)	1.05211 (14)	0.74416 (12)	0.0409 (3)	
O3	0.51842 (12)	0.50532 (17)	0.96186 (14)	0.0482 (3)	
O4	0.34354 (16)	0.59370 (17)	1.11319 (11)	0.0504 (3)	
O5	0.14543 (15)	0.01552 (16)	1.26375 (12)	0.0472 (3)	
N1	0.19095 (16)	1.02862 (18)	0.54219 (13)	0.0365 (3)	
N2	0.30128 (13)	0.35864 (15)	0.97875 (12)	0.0310 (3)	
N3	0.29017 (12)	0.32938 (16)	0.60656 (12)	0.0285 (3)	
C1	0.17016 (14)	0.79352 (17)	0.70936 (12)	0.0241 (3)	
C2	0.16170 (15)	0.64755 (17)	0.66712 (13)	0.0262 (3)	
H2	0.1130	0.6168	0.5884	0.031*	
C3	0.29337 (15)	0.62151 (18)	0.85939 (14)	0.0269 (3)	
C4	0.23410 (14)	0.54569 (18)	0.75421 (13)	0.0248 (3)	
C5	0.23691 (15)	0.37508 (18)	0.73479 (14)	0.0262 (3)	
H5	0.1394	0.3386	0.7368	0.031*	
C6	0.31904 (17)	0.29191 (18)	0.84604 (15)	0.0312 (3)	
H6A	0.2895	0.1844	0.8473	0.037*	
H6B	0.4182	0.2939	0.8264	0.037*	
C7	0.15834 (18)	0.3485 (2)	1.02768 (17)	0.0410 (4)	
H7A	0.0916	0.3777	0.9557	0.049*	
H7B	0.1486	0.4212	1.1012	0.049*	
C8	0.12434 (19)	0.1893 (2)	1.07530 (16)	0.0409 (4)	
H8A	0.0237	0.1818	1.0872	0.049*	
H8B	0.1489	0.1149	1.0069	0.049*	
C9	0.2000 (2)	0.1493 (2)	1.20395 (16)	0.0414 (4)	
H9A	0.2988	0.1329	1.1873	0.050*	
H9B	0.1935	0.2355	1.2661	0.050*	
C10	0.1941 (2)	−0.1233 (3)	1.2128 (2)	0.0497 (5)	
H10A	0.1556	−0.1381	1.1229	0.074*	
H10B	0.1652	−0.2072	1.2690	0.074*	
H10C	0.2950	−0.1209	1.2114	0.074*	
C11	0.42904 (19)	0.3825 (3)	0.57763 (18)	0.0499 (5)	
H11A	0.4982	0.3234	0.6305	0.060*	
H11B	0.4386	0.4905	0.6033	0.060*	
C12	0.4563 (3)	0.3657 (5)	0.4339 (2)	0.0923 (12)	
H12A	0.4480	0.2586	0.4087	0.138*	
H12B	0.5497	0.4016	0.4175	0.138*	
H12C	0.3890	0.4258	0.3816	0.138*	
H3	0.2359 (17)	0.369 (2)	0.5477 (16)	0.038 (5)*	
H1A	0.228 (2)	1.1141 (16)	0.566 (2)	0.044 (6)*	
H1B	0.174 (2)	1.024 (3)	0.4577 (10)	0.046 (5)*	

### Atomic displacement parameters (Å^2^)

**Table:**

	*U*^11^	*U*^22^	*U*^33^	*U*^12^	*U*^13^	*U*^23^
S1	0.03112 (17)	0.02019 (17)	0.02929 (17)	0.00014 (14)	−0.00565 (13)	0.00508 (14)
S2	0.04226 (19)	0.01977 (18)	0.02583 (16)	−0.00143 (14)	−0.01218 (13)	−0.00112 (14)
S3	0.0398 (2)	0.0277 (2)	0.02842 (17)	0.00293 (16)	−0.01163 (13)	0.00436 (15)
O1	0.0434 (7)	0.0388 (7)	0.0599 (8)	−0.0078 (6)	−0.0284 (6)	0.0154 (6)
O2	0.0534 (7)	0.0300 (7)	0.0398 (6)	0.0094 (5)	0.0096 (5)	0.0022 (5)
O3	0.0345 (6)	0.0476 (8)	0.0612 (8)	0.0005 (6)	−0.0145 (5)	0.0135 (7)
O4	0.0838 (10)	0.0383 (7)	0.0280 (6)	0.0073 (7)	−0.0134 (6)	−0.0016 (5)
O5	0.0716 (9)	0.0387 (7)	0.0326 (6)	0.0081 (7)	0.0188 (6)	0.0074 (6)
N1	0.0563 (8)	0.0259 (8)	0.0274 (6)	−0.0088 (7)	0.0012 (5)	0.0046 (6)
N2	0.0382 (6)	0.0250 (7)	0.0297 (6)	0.0047 (5)	0.0010 (5)	0.0062 (5)
N3	0.0306 (6)	0.0254 (7)	0.0291 (6)	−0.0013 (5)	−0.0040 (4)	−0.0020 (5)
C1	0.0283 (6)	0.0219 (8)	0.0216 (6)	−0.0007 (5)	−0.0055 (5)	0.0033 (5)
C2	0.0324 (7)	0.0211 (7)	0.0246 (6)	−0.0015 (5)	−0.0064 (5)	0.0004 (6)
C3	0.0328 (7)	0.0196 (8)	0.0276 (7)	0.0021 (6)	−0.0074 (5)	0.0031 (6)
C4	0.0279 (6)	0.0205 (7)	0.0257 (6)	0.0010 (5)	−0.0037 (5)	0.0025 (6)
C5	0.0280 (6)	0.0202 (7)	0.0301 (7)	0.0013 (5)	−0.0034 (5)	0.0005 (6)
C6	0.0394 (7)	0.0217 (8)	0.0322 (7)	0.0059 (6)	−0.0008 (6)	0.0026 (6)
C7	0.0414 (8)	0.0419 (11)	0.0402 (8)	0.0084 (7)	0.0087 (6)	0.0116 (7)
C8	0.0458 (9)	0.0453 (11)	0.0317 (7)	−0.0054 (8)	0.0035 (6)	0.0066 (8)
C9	0.0549 (10)	0.0368 (10)	0.0327 (8)	0.0009 (8)	0.0042 (7)	0.0026 (7)
C10	0.0651 (12)	0.0380 (10)	0.0462 (10)	0.0054 (9)	0.0051 (8)	−0.0013 (9)
C11	0.0390 (9)	0.0666 (13)	0.0444 (9)	−0.0152 (9)	0.0070 (7)	−0.0117 (10)
C12	0.0653 (15)	0.153 (4)	0.0599 (14)	−0.0316 (19)	0.0227 (11)	−0.0246 (18)

### Geometric parameters (Å, º)

**Table:**

S1—O1	1.4338 (12)	C4—C5	1.517 (2)
S1—O2	1.4346 (13)	C5—C6	1.540 (2)
S1—N1	1.5834 (14)	C5—H5	1.0000
S1—C1	1.7600 (15)	C6—H6A	0.9900
S2—C1	1.7205 (13)	C6—H6B	0.9900
S2—C3	1.7219 (16)	C7—C8	1.524 (3)
S3—O4	1.4256 (14)	C7—H7A	0.9900
S3—O3	1.4274 (14)	C7—H7B	0.9900
S3—N2	1.6349 (15)	C8—C9	1.513 (2)
S3—C3	1.7592 (14)	C8—H8A	0.9900
O5—C10	1.416 (2)	C8—H8B	0.9900
O5—C9	1.436 (2)	C9—H9A	0.9900
N1—H1A	0.866 (10)	C9—H9B	0.9900
N1—H1B	0.866 (9)	C10—H10A	0.9800
N2—C6	1.484 (2)	C10—H10B	0.9800
N2—C7	1.493 (2)	C10—H10C	0.9800
N3—C11	1.466 (2)	C11—C12	1.497 (3)
N3—C5	1.4731 (19)	C11—H11A	0.9900
N3—H3	0.856 (9)	C11—H11B	0.9900
C1—C2	1.357 (2)	C12—H12A	0.9800
C2—C4	1.4245 (19)	C12—H12B	0.9800
C2—H2	0.9500	C12—H12C	0.9800
C3—C4	1.365 (2)		
			
O1—S1—O2	120.25 (9)	N2—C6—H6A	108.9
O1—S1—N1	108.78 (8)	C5—C6—H6A	108.9
O2—S1—N1	109.28 (8)	N2—C6—H6B	108.9
O1—S1—C1	105.28 (7)	C5—C6—H6B	108.9
O2—S1—C1	105.47 (7)	H6A—C6—H6B	107.7
N1—S1—C1	106.95 (8)	N2—C7—C8	112.05 (14)
C1—S2—C3	89.70 (7)	N2—C7—H7A	109.2
O4—S3—O3	118.92 (9)	C8—C7—H7A	109.2
O4—S3—N2	109.63 (8)	N2—C7—H7B	109.2
O3—S3—N2	108.14 (8)	C8—C7—H7B	109.2
O4—S3—C3	109.63 (8)	H7A—C7—H7B	107.9
O3—S3—C3	108.34 (8)	C9—C8—C7	112.58 (16)
N2—S3—C3	100.62 (7)	C9—C8—H8A	109.1
C10—O5—C9	114.96 (14)	C7—C8—H8A	109.1
S1—N1—H1A	118.3 (14)	C9—C8—H8B	109.1
S1—N1—H1B	118.6 (15)	C7—C8—H8B	109.1
H1A—N1—H1B	112 (2)	H8A—C8—H8B	107.8
C6—N2—C7	114.80 (13)	O5—C9—C8	112.38 (16)
C6—N2—S3	110.94 (10)	O5—C9—H9A	109.1
C7—N2—S3	116.48 (11)	C8—C9—H9A	109.1
C11—N3—C5	116.46 (13)	O5—C9—H9B	109.1
C11—N3—H3	105.9 (14)	C8—C9—H9B	109.1
C5—N3—H3	106.0 (14)	H9A—C9—H9B	107.9
C2—C1—S2	113.32 (10)	O5—C10—H10A	109.5
C2—C1—S1	126.55 (10)	O5—C10—H10B	109.5
S2—C1—S1	119.95 (9)	H10A—C10—H10B	109.5
C1—C2—C4	112.31 (12)	O5—C10—H10C	109.5
C1—C2—H2	123.8	H10A—C10—H10C	109.5
C4—C2—H2	123.8	H10B—C10—H10C	109.5
C4—C3—S2	113.72 (11)	N3—C11—C12	111.18 (16)
C4—C3—S3	121.46 (12)	N3—C11—H11A	109.4
S2—C3—S3	124.60 (9)	C12—C11—H11A	109.4
C3—C4—C2	110.94 (13)	N3—C11—H11B	109.4
C3—C4—C5	125.25 (13)	C12—C11—H11B	109.4
C2—C4—C5	123.73 (13)	H11A—C11—H11B	108.0
N3—C5—C4	113.21 (12)	C11—C12—H12A	109.5
N3—C5—C6	109.05 (12)	C11—C12—H12B	109.5
C4—C5—C6	112.84 (13)	H12A—C12—H12B	109.5
N3—C5—H5	107.1	C11—C12—H12C	109.5
C4—C5—H5	107.1	H12A—C12—H12C	109.5
C6—C5—H5	107.1	H12B—C12—H12C	109.5
N2—C6—C5	113.55 (12)		

### Hydrogen-bond geometry (Å, º)

**Table:**

*D*—H···*A*	*D*—H	H···*A*	*D*···*A*	*D*—H···*A*
N1—H1*B*···O5^i^	0.87 (1)	1.98 (1)	2.841 (2)	177 (2)
N1—H1*A*···N3^ii^	0.87 (1)	2.03 (1)	2.886 (2)	171 (2)
N3—H3···O1^iii^	0.86 (1)	2.26 (1)	3.042 (2)	151 (2)

Symmetry codes: (i) *x*, *y*+1, *z*−1; (ii) *x*, *y*+1, *z*; (iii) −*x*, *y*−1/2, −*z*+1.
